# Solubility, porosity and fluid uptake of calcium silicate-based cements

**DOI:** 10.1590/1678-7757-2017-0465

**Published:** 2018-05-11

**Authors:** Fernanda Ferrari Esteves Torres, Juliane Maria Guerreiro-Tanomaru, Roberta Bosso-Martelo, Gisselle Moraima Chavez-Andrade, Mario Tanomaru

**Affiliations:** 1 Universidade Estadual Paulista (UNESP), Faculdade de Odontologia de Araraquara, Departamento de Odontologia Restauradora, Araraquara, São Paulo, Brasil.; 2 Universidade Federal da Bahia, Faculdade de Odontologia, Departamento de Clínica Odontologia, Salvador, Bahia, Brasil.

**Keywords:** Physical and chemical properties, Porosity, Solubility, X-Ray microtomography

## Abstract

**Objective:**

To evaluate the absorption/fluid uptake, solubility and porosity of White mineral trioxide aggregate (MTA) Angelus, Biodentine (BIO), and zinc oxide-eugenol (ZOE).

**Material and Methods:**

Solubility was evaluated after immersion in distilled water for 7 and 30 days. Porosity was evaluated using digital inverted microscope, scanning electron microscope (SEM) and micro-computed tomography (micro-CT). For the fluid uptake test, specimens were immersed in Hank's balanced salt solution (HBSS) for 1, 7, 14 and 28 days. Fluid absorption, solubility and porosity of the materials were measured after each period. Statistical evaluation was performed using one-way analysis of variance (ANOVA) and Tukey tests, with a significance level at 5%.

**Results:**

After 7 and 30 days, BIO showed the highest solubility (p<0.05). All methods demonstrated that MTA had total porosity higher than BIO and ZOE (p<0.05). Micro-CT analysis showed that MTA had the highest porosity at the initial period, after its setting time (p<0.05). After 7 and 30 days, ZOE had porosity lower than MTA and BIO (p<0.05). Absorption was similar among the materials (p>0.05), and higher fluid uptake and solubility were observed for MTA in the fluid uptake test (p<0.05).

**Conclusions:**

BIO had the highest solubility in the conventional test and MTA had higher porosity and fluid uptake. ZOE had lower values of solubility, porosity and fluid uptake. Solubility, porosity and fluid uptake are related, and the tests used provided complementary data.

## Introduction

Root repair cements must have appropriate physicochemical properties. It should have low solubility in tissue fluids^7^ ^,^ ^24^, since the dissolution of materials may allow leakage, leading to treatment failure^7^ ^,^ ^10^. Solubility is evaluated using standardized samples of material, which are weighed before and after immersion in distilled water as defined by the ISO 6876:2002^16^ or ANSI/ADA specification No. 57^1^.

Another important property of root repair cements is the porosity, which can affect the physical properties and, consequently, its behavior within its environment^4^. Standardized tests are used to assess this material property and alternative methods have been suggested. Therefore, to evaluate physical properties using different methods is relevant. The porosity of endodontic materials may be evaluated using microscopy^5^. However, this technique produces two-dimensional data and may be inexact to show the internal porosity of the material^26^. Therefore, micro-computed tomography (micro-CT) may be used as an alternative method for evaluating porosity and distribution of the size of pores within the material^23^. The porosity may be associated with solubility^3^ ^,^ ^8^ ^,^ ^27^, suggesting that a porous material has higher solubility.

As another alternative assay for materials, the fluid uptake test evaluates absorption of fluid (the quantity of fluid adsorbed by the material), solubility (quantity of substance dissolved in a quantity of solvent) and porosity (empty spaces). This test is not part of the ISO or ANSI/ADA standards, but allows the testing of both solubility and porosity together with absorption^15^. Moreover, the use of HBSS solution for immersion of materials provides greater similarity to clinical application^15^.

Several root repair and root-end filling cements are available for clinicians; and although the clinical performances considering physicochemical and biological properties of these materials have been evaluated, no material can be considered ideal^24^. Mineral Trioxide Aggregate (MTA) is a calcium silicate-based cement developed by Torabinejad, et al.^24^ (1993) for the treatment of root perforations, root-end filling, and other indications. Biodentine (BIO) is a calcium silicate-based reparative and restorative cement with indications similar to MTA^19^, with enhanced properties, such as quick setting time^15^. Zinc oxide-eugenol (ZOE) may be used as a root-end filling material, with solubility similar to MTA after 30 days of immersion in distilled water^11^. However, these materials may behave differently at clinical settings, depending on their properties.

The aim of this study was to evaluate the solubility, porosity, and fluid uptake of root repair cements, using conventional and complementary methods.

## Material and methods

**Figure 1:**
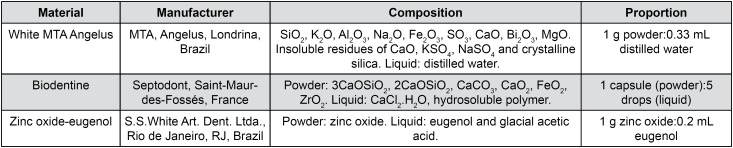
Root-end filling material, manufacturers, and proportions used

White MTA Angelus (MTA, Angelus, Londrina, PR, Brazil), Biodentine (BIO, Septodont, Saint-Maur-des-Fossés, France) and zinc oxide-eugenol (ZOE, S.S. White Art. Dent. Ltda., Rio de Janeiro, RJ, Brazil) were used in the proportions described in Figure 1. These materials were manipulated and subjected to solubility, porosity and absorption (*i.e.*, fluid uptake) analyses.

### Solubility

The solubility test was performed based on a previous study^6^. Cylindrical molds of 1.5 mm in thickness and 7.75 mm in internal diameter (n=6) were filled with each cement and a nylon thread. The samples were placed in an oven with 95% relative humidity at 37°C for 24 hours. The test specimens were removed from the molds and weighed three times, each in an analytical balance with accuracy of ± 0.0001 g (AR2140, Toledo do Brasil Indústria de Balanças Ltda., São Bernardo do Campo, SP, Brazil), and the mean reading was recorded. The samples were placed in plastic receptacles with lids containing 7.5 mL of distilled and deionized water, suspended by nylon threads attached to the containers. The receptacles remained in the oven at 37°C for 7 days. After this period, the test specimens were removed from the distilled water, dried and placed in a dehumidifying chamber. The mass was measured before and after immersion of the samples in distilled water, and every 24 hours thereafter, until attaining mass stabilization. New samples were prepared and kept immersed in distilled water for 30 days. The mass loss was expressed as a percentage of the original mass.

### Porosity analysis using inverted optical microscope

The methodology to evaluate porosity was based on a previous study^3^. The microstructure of the materials was observed using a digital inverted microscope (MIC-D Olympus, Philippines) on rectangular specimens (n=6) measuring 8×10 mm and 5 mm high. The specimens were prepared and then stored for 24 hours at 37°C and 95% humidity. The specimens were then unmolded and stored in distilled water for 7 days, after which they were sectioned in half along their cross section with a microtome cutter (IsoMet 1000, Buehler Ltd, Lake Bluff, IL, USA). They were subsequently polished using fine grit of silicon carbide abrasive paper numbers 320 and 600, and rinsed with distilled water after the polishing procedure. The specimen surfaces were observed with the digital inverted microscope at 50x and 200x magnification. The images were captured and analyzed qualitatively and quantitatively to search for the presence of pores. Quantitative analysis of pores was performed using the software ImageTool version 3.0 (UTHSCSA, San Antonio, TX, USA). The surface of the material was divided into four parts, and each part was individually analyzed at the two magnifications used.

### Porosity analysis using micro-CT

Porosity analysis using micro-CT was based on previous study^23^. Cylindrical test specimens measuring 4±0.1 mm in thickness and 7±0.1 mm in diameter were fabricated (n=6). The materials were spatulated, placed in the molds and stored for 24 hours at 37°C and 95% humidity.

The samples were examined using micro-CT (SkyScan 1176, Bruker microCT, Kontich, Belgium) after setting (initial time) and after immersion in distilled water for the time intervals of 7 and 30 days. The initial porosity of the materials and after contact with the aqueous solution were evaluated. The scanning parameters were: voltage at 80 kV, current at 313 μA, pixel size of 9 μm and 360° rotation using a Cu+Al filter. These images were used for quantitative analysis of the samples, allowing the porosity of the material to be calculated in mm^3^ and percentage. Reconstruction of the images was performed using NRecon software (V1.6.4,7; Bruker microCT, Kontich, Belgium). The correction parameters for smoothening, beam hardening and ring artefacts were defined for each material (parameters for MTA were 1 for smoothing, 75 for beam hardening correction and 8 for ring artefacts; for ZOE, they were 9, 90 and 8, respectively; and for BIO, they were 1, 90 and 8). The same parameters were used for the same material at different periods. The reconstructed images were superposed at the different periods and saved in the coronal, sagittal and transaxial planes using the Data Viewer program (V1.5.2.4; Bruker microCT, Kontich, Belgium). The images were analyzed using CTAn software (V1.11.8; Bruker MicroCT, Kontich, Belgium). Open, closed and total porosity were evaluated, in which the closed pores represent empty spaces surrounded by material; and open pores are in contact with the external surface.

### Surface analysis using scanning electron microscope (SEM)

For analysis of the surface morphology of the different experimental groups, the materials were manipulated and inserted into cylindrical molds with 6 mm in diameter and 12 mm high (n=6). Specimens were kept in an oven at 37°C and immersed in distilled water for 28 days. After this period, the test specimens were dried with absorbent paper and kept in a vacuum desiccator containing silica for 24 hours. The specimens were embedded in resin and polished in an automatic polishing machine (EcoMet 250 Grinder/Polisher Family, Illinois, USA). After being dried again, the specimens were placed on stubs, coated with carbon, and examined using a scanning electron microscope (JEOL JSM-6610LV, Tokyo, Japan) at three magnifications (50x, 500x, and 1000x) in secondary backscattered electron mode. All analyses were performed at 18 kV and SS 68. The images obtained were subjected to qualitative evaluation regarding the porosity.

### Fluid uptake, absorption, solubility and porosity

This test was based on a previous study^15^. The specimens were prepared measuring 15 mm in diameter and 1±0.1 mm thickness (n=6). After setting in an oven at 37°C for 24 hours, the mass was recorded (m1). The mean diameter of each specimen and the thickness of each specimen were measured at an accuracy of ±0.01 mm used to calculate the volume (V) of each disc. The specimens were then immersed uprightly in 10 mL of HBSS. After 1 day of immersion, the specimens were removed, dried with filter paper and weighed (m). The fluid uptake of each sample was calculated using the equation: Fluid uptake (%)=m-m1/Vx100. This process was repeated after time intervals of 1, 7, 14 and 28 days. Variation in fluid uptake over time was noted. After 28 days, the mass of specimens (totally saturated with water) was measured (m2). The specimens were then dried by placing them in a vacuum desiccator for 24 hours, using silica gel as desiccant, to constant mass (m3). Absorption and solubility were calculated. The water absorption for each sample was calculated using the equation: Absorption (%)=m2-m3/Vx100. Solubility was calculated according to the equation: Solubility (%)=m1-m3/Vx100. The porosity of each sample was calculated according to the equation: Porosity (%)=[(m2/m1)-1]x100. The mass of HBSS absorbed through the pores of each specimen was quantified based on the Archimedes' Principle. Measurement of the difference in mass (g) between each sample when dry and when submersed in solution was expressed as the volume of the pores in each sample.

### Statistical analysis

Data obtained for all the tests were submitted to normality test, and then to the parametric one-way analysis of variance (ANOVA) statistical test and the Tukey multiple comparison test, with significance level at 5%.

## Results

### Solubility

At 7 days, BIO showed the highest solubility (p<0.05), followed by ZOE. The value was lower for MTA, which gained weight (p<0.05). After 30 days, MTA and ZOE had comparable solubility (p>0.05), which was lower than that of BIO (p<0.05) (Table 1).

**Table 1:** Mean and standard deviation of the results of solubility (%) and porosity (number of pores) of root repair cements evaluated after immersion in distilled water

Materials/Tests	White MTA Angelus	BIO	ZOE
Porosity	136(±36.61)^a^	71.50(±32.23)^b^	48.69(±21.30)^b^
Solubility 7 days (mass loss)	-1.36(±0.69)^c^	6.72(±0.37)^a^	3.27(±0.24)^b^
Solubility 30 days (mass loss)	4.12(±2.06)^b^	7.34(±2.08)^a^	4.65(±0.64)^b^

a,b,c Different letters indicate statistically significant difference between experimental groups (p<0.05)

MTA: mineral trioxide aggregate

BIO: Biodentine

ZOE: Zinc oxide-eugenol

### Porosity analysis using digital inverted microscope

**Figure 2:**
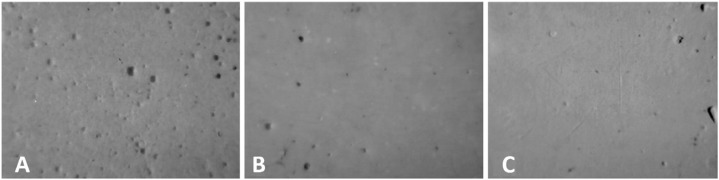
Porosity observed for (A) White mineral trioxide aggregate (MTA) Angelus, (B) Biodentine (BIO), and (C) Zinc oxide-eugenol (ZOE) cements using microscopic evaluation at 50x magnification

The number of pores was higher for MTA (p<0.05), and there was no statistically significant difference between BIO and ZOE (p>0.05) (Table 1). Representative images captured at 50x magnification of all materials evaluated are shown in Figure 2.

### Porosity analysis using micro-CT

**Figure 3:**
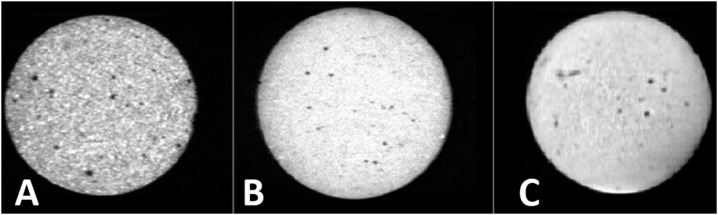
Microtomographic images captured with the CTAn program, representative of the porosity for (A) White mineral trioxide aggregate (MTA) Angelus, (B) Biodentine (BIO), and (C) Zinc oxide-eugenol (ZOE) cements at the initial period

The porosity observed in micro-CT is represented in Table 2, and representative images of the porosity of the materials are illustrated in Figure 3. For the open and the total porosities, MTA had comparable values at the baseline and 7-day time intervals (p>0.05), and higher values after 30 days (p<0.05). BIO had an increase in the values of open and total porosities after immersion in water (p<0.05), and ZOE maintained comparable values in the three time intervals (p>0.05). At the baseline, MTA had the highest porosity values (p<0.05), and after 7 and 30 days, MTA and BIO showed comparable values (p>0.05) and higher than ZOE (p<0.05). For closed porosity, all three materials maintained their values in the three time intervals (p>0.05); in the initial time interval, the three materials showed a comparable result (p>0.05) and after 7 and 30 days, MTA showed values comparable to those of BIO (p>0.05) and higher than those of ZOE (p<0.05).

**Table 2:** Mean and standard deviation of micro-CT data for open, closed, and total porosity (%) of the materials at initial time, and after 7 and 30 days of immersion in distilled water

Materials/Tests	White MTA Angelus	BIO	ZOE
Initial open porosity	14.67(±4.67)^a^,^B^	5.91(±1.12)^b^,^C^	6.34(±0.83)^b^,^A^
Open porosity at 7 days	14.02(±4.34)^a^,^B^	16.28(±2.40)^a^,^B^	7.30(±0.83)^b^,^A^
Open porosity at 30 days	22.18(±4.67)^a^,^A^	25.87(±3.00)^a^,^A^	7.39(±1.01)^b^,^A^
Initial closed porosity	0.46(±0.20)^a^,^A^	0.40(±0.07)^a^,^B^	0.26(±0.09)^a^,^C^
Closed porosity at 7 days	0.39(±0.13)^a^,^A^	0.26(±0.12)a,b,^B^	0.20(±0.05)^b^,^C^
Closed porosity at 30 days	0.42(±0.20)^a^,^A^	0.27(±0,09)a,b,^B^	0.20(±0.06)^b^,^C^
Initial total porosity	15.19(±4.83)^a^,^B^	6.36(±1.03)^b^,^C^	6.71(±1.01)^b^,^A^
Total porosity at 7 days	14.48(±4.60)^a^,^B^	16.59(±2.23)^a^,^B^	7.47(±0.84)^b^,^A^
Total porosity at 30 days	22.50(±4.97)^a^,^A^	26.27(±2.98)^a^,^A^	7.68(±0.86)^b^,^A^

a,b,c Different lowercase letters on the same line indicate statistically significant difference between experimental groups in the same period (p<0.05)

A,B Different capital letters in the same column indicate statistically significant difference between the same groups in the different periods (p<0.05)

MTA: mineral trioxide aggregate

BIO: Biodentine

ZOE: Zinc oxide-eugenol

### Porosity analysis using SEM

**Figure 4:**
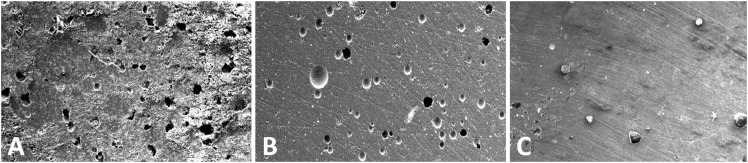
Porosity of (A) White mineral trioxide aggregate (MTA) Angelus, (B) Biodentine (BIO), and (C) Zinc oxide-eugenol (ZOE) cements by scanning electron microscopy (SEM) evaluation at 50x magnification

Qualitative analysis by scanning electron microscope showed a higher porosity for MTA (Figure 4), because the surface of this cement was irregular and filled with empty spaces. BIO showed a regular surface with some porosity. The lowest porosity was observed for ZOE.

### Fluid uptake

MTA had the highest fluid uptake values at all periods (p<0.05), followed by BIO; while lower values were observed for ZOE. There was no statistically significant difference between the absorption for the examined materials (p>0.05). Porosity was higher for MTA and lower for BIO (p<0.05) (Table 3).

**Table 3:** Mean and standard deviation of the results of fluid uptake, absorption, porosity and solubility (%) after immersion in Hank's balanced salt solution (HBSS)

Materials/Tests	White MTA Angelus	BIO	ZOE
Fluid uptake after 24 h	0.02276(±0.0017)^a^	0.01099(±0.0021)^b^	0.00261(±0.0005)^c^
Fluid uptake after 7 days	0.02326(±0.0018)^a^	0.009463(±0.0050)^b^	0.00125(±0.0096)^c^
Fluid uptake after 14 days	0.0242(±0.0023)^a^	0.01316(±0.0026)^b^	0.001853(±0.0015)^c^
Fluid uptake after 28 days	0.03517(±0.0021)^a^	0.001787(±0.0053)^c^	0.01340(±0.0035)^b^
Absorption	0.0109(±0.0010)^a^	0.0139 (±0.0046)^a^	0.0097(±0.0016)^a^
Porosity	25.06(±1.708)^a^	0.1765(±1.473)^c^	6.808(±0.970)^b^
Solubility	0.0242(±0.0020)^a^	0.0123(±0.0005)^b^	0.0035(±0.0021)^c^

a,b,c Different letters indicate statistically significant difference between experimental groups (p<0.05)

MTA: mineral trioxide aggregate

BIO: Biodentine

ZOE: Zinc oxide-eugenol

## Discussion

In accordance with the ISO standard 6876/2002, the solubility is evaluated after a period of 24 hours. However, longer periods may be used^2^ ^,^ ^25^. The periods used in this study (7 and 30 days) can be used for comparison with the results of fluid uptake test. At 7 and 30 days, solubility was higher for BIO, in agreement with a previous study^10^.

The fluid uptake test also evaluates the solubility of materials. However, when compared to the solubility test, there are differences with the diameter of the specimens, time and immersion solution, and dehydration after removal from the solution. The fluid uptake test allows the analysis of the absorption of fluid, solubility, and porosity of the materials in a single test. In general, this study showed higher values of fluid uptake for MTA, followed by BIO, and lower values for ZOE, without difference in absorption for the tested materials. Regarding the solubility, in the fluid uptake test, the materials were immersed in the HBSS solution for 28 days, whereas the conventional test analyses were performed after immersion in distilled water for 7 and 30 days. MTA showed the greatest increase in solubility after 30 days, suggesting association with the fluid uptake test, in which the highest solubility was observed for MTA after 28 days. Kaup, et al.^17^ (2015) evaluated the solubility of ProRoot MTA (Dentsply Tulsa Dental, Tulsa, OK, USA) and BIO in distilled water and phosphate buffered saline (PBS) at different periods. When immersed in distilled water, BIO was significantly more soluble than ProRoot MTA for all evaluated periods. However, the higher solubility of BIO decreased in PBS, in agreement with this study, probably due to the precipitated surface crystals, which is released from di- and tricalcium silicate-containing materials and avoids a further increase of solubility^22^.

A previous investigation of the fluid uptake of three cements [Bioaggregate, BIO and intermediate restorative material (IRM)] and a calcium silicate-based experimental cement^15^, demonstrated that BIO had the lowest fluid uptake values and was similar to IRM (based on zinc oxide-eugenol), with values similar to observed in this study. Moreover, the absorption and solubility results were similar for BIO and IRM which agrees with the results of the current study. Previous studies evaluated the fluid uptake of MTA (White MTA, Dentsply; Tulsa Dental Products, Tulsa, OK, USA), which showed an increase in weight throughout the period of immersion in HBSS^3^ ^,^ ^5^, as reported in this study. The fluid uptake test was also used to evaluate calcium silicate-based materials after immersion in distilled water for 24 hours. The highest solubility and porosity values were observed for White MTA Angelus^14^, in agreement with the results of this study.

Porosity occurs because of spaces in the non-hydrated cement^18^, and the porosity and solubility of materials may affect their stability, integrity, and durability^21^. The porosity may be visually determined by observing the size and distribution of pores in the polished surface of cements. However, it is not a precise method, due to its qualitative evaluation^18^. In this study, the captured images using the digital inverted microscope at 50x and 200x magnification were transferred to the ImageTool 3.0 software (UTHSCSA, San Antonio, TX, USA), allowing the counting of the pores in the cements and the conduction of a quantitative comparison.

The cutting blade used to prepare specimens for surface analysis of materials via SEM may change the porosities, which can influence on the measurement of the number and size of pores^26^. The surface evaluation of materials using micro-CT allowed nondestructive testing and has been used for evaluating the porosity of different materials^13^ ^,^ ^23^. Micro-CT provides data on open and closed porosity, in which the closed pores represent empty spaces surrounded by material; and open pores are those in contact with the external surface.

According to the manufacturer (Bruker MicroCT, Kontich, Belgium), the CTAn software allows the porosity analysis of any material. In this study, the specimens were scanned in a high pixel size (9 μm), which increased the quality and reliability of images.

Solubility and porosity have been linked to each other. The solubility observed for MTA may be associated with the presence of bismuth oxide as radiopacifier, which increases the porosity of the cement, decreasing its mechanical stability and increasing its solubility^3^ ^,^ ^8^. The evaluation of the solubility and porosity of MTA, using different powder/water ratios, showed a correlation between the two properties, which grew with the increase of water in the mixture^12^.

MTA had greater fluid absorption in the fluid uptake test, which may favor the expansion of the material. However, absorption may favor an increase in porosity^4^, which justifies the higher porosity values found for MTA in the fluid uptake test, when examined under a microscope, by SEM and in the initial period via micro-CT. Considering the similarities between Portland Cement and MTA, the increase in weight after 7 days in distilled water, probably occurs due the empty pores and capillaries, which are rechargeable, and the newly captured water will then participate in the hydration process of these cements, increasing their weight^12^. ZOE showed solubility higher than MTA at 7 days and comparable values at 30 days, in agreement with previous studies^11^ ^,^ ^25^. This solubility can be related to the setting reaction of zinc oxide-eugenol. Zinc is a metal likely to form a chelate (zinc eugenolate) and eugenol has a replaceable hydrogen and a nearby donor in the oxygen^9^. When ZOE is immersed in water, eugenol is eluted by leaching and a progressive decomposition of the zinc eugenolate matrix occurs, causing a disintegration of the material^28^. Low porosity and fluid uptake were observed for ZOE, according to Camilleri and Mallia^5^ (2011), who evaluated a sealer based on zinc oxide-eugenol (Pulp canal sealer, SybronEndo Corporation, Orange, CA, USA) and MTA. The authors observed a uniform surface with homogenous distribution of the particles in the pulp canal sealer and the highest degree of porosity in MTA.

BIO has a reduced particle size^19^, in addition to the use of additives to reduce porosity^5^. Another important aspect is the presence of zirconium oxide as radiopacifier that has a smaller particle size and greater filling of spaces occupied by the calcium silicate^20^. These factors may justify the lower porosity values observed for BIO when compared with MTA in the fluid uptake tests and microscopy. Moreover, BIO has polycarboxylate in its composition to favor the manipulation and insertion of cement. However, it has a surfactant effect that may increase the solubility of the material^10^. By increasing solubility, there may be greater disintegration of the material, justifying the increase in micro-CT results of open and total porosity after immersion of specimens in distilled water.

## Conclusions

BIO showed the highest solubility in conventional test. MTA had higher fluid uptake and total porosity values when evaluated by microscope, SEM, fluid uptake test and micro-CT in the initial period. ZOE had lower values of solubility, porosity and fluid uptake. Solubility, porosity and fluid uptake are related, and the conventional and modern tests provided complementary information and results.
